# Development of a Hybrid Atomic Force Microscopic Measurement System Combined with White Light Scanning Interferometry

**DOI:** 10.3390/s120100175

**Published:** 2011-12-27

**Authors:** Tong Guo, Siming Wang, Dante J. Dorantes-Gonzalez, Jinping Chen, Xing Fu, Xiaotang Hu

**Affiliations:** State Key Laboratory of Precision Measuring Technology and Instruments, Tianjin University, Weijin Road, No.92, Tianjin 300072, China; E-Mails: siming_w@tju.edu.cn (S.W.); dante_dorantes@hotmail.com (D.J.D.-G.); chenjinping@tju.edu.cn (J.C.); xingfu@tju.edu.cn (X.F.); xthu@tju.edu.cn (X.H.)

**Keywords:** atomic force microscope, self-sensing tuning fork probe, white light scanning interferometry, large-range positioning platform, hybrid measurement

## Abstract

A hybrid atomic force microscopic (AFM) measurement system combined with white light scanning interferometry for micro/nanometer dimensional measurement is developed. The system is based on a high precision large-range positioning platform with nanometer accuracy on which a white light scanning interferometric module and an AFM head are built. A compact AFM head is developed using a self-sensing tuning fork probe. The head need no external optical sensors to detect the deflection of the cantilever, which saves room on the head, and it can be directly fixed under an optical microscopic interferometric system. To enhance the system’s dynamic response, the frequency modulation (FM) mode is adopted for the AFM head. The measuring data can be traceable through three laser interferometers in the system. The lateral scanning range can reach 25 mm × 25 mm by using a large-range positioning platform. A hybrid method combining AFM and white light scanning interferometry is proposed to improve the AFM measurement efficiency. In this method, the sample is measured firstly by white light scanning interferometry to get an overall coarse morphology, and then, further measured with higher resolution by AFM. Several measuring experiments on standard samples demonstrate the system’s good measurement performance and feasibility of the hybrid measurement method.

## 1. Introduction

With the development of micro/nanometer manufacturing technologies, the structure and number of devices with micro/nanometer dimensions have increased greatly, which imposes a higher demand on micro/nanometer dimensional measurement technologies. Atomic force microscopy (AFM) is one of the most important micro/nanometer dimensional measurement methods. Since its invention in 1986 [1], AFM has become an indispensible tool for topographical characterization with nano- and sub-nanometer resolution.

Compared with other micro/nanometer dimensional measurement methods, such as stylus profilometry and optical microscopy [2], the biggest advantage of AFM is its high resolution, reaching in both in lateral and vertical direction, up to nano- and sub-nanometer ranges; however, there are also some shortcomings of AFM in measuring efficiency and range.

Due to the use of a point-by-point scanning measuring mode, compared with other optical measuring methods, the measuring efficiency of AFM is obviously limited. In this regard, there are two main approaches to improve AFM’s measuring efficiency. One is intended to enhance the measuring speed of the AFM from both the hardware and software points of view [3], such as using scanners with higher resonant frequency and servo controllers with higher dynamic response [4] or adopting feedback control schemes with higher speed. The other approach is to combine AFM with other measuring methods, like optical measuring methods.

Unlike AFM, optical measuring methods use the field measuring mode meaning that a sample surface is detected in a large area at the same time (millions of points), therefore, the measuring efficiency can be greatly improved. In some recent studies, some methods based on the combination of AFM and optical measuring methods have been researched. Jobin integrated an optical interference measuring head into a commercial AFM, but owing to the limitation of optical interference objective’s working distance, the AFM can’t be directly fixed under an optical measuring head. When using AFM, the optical interference objective must be moved away [5]. Tyrrell combined an AFM head with an optical microscope [6]. When using a home-made quartz tuning fork probe, the AFM head has a self-sensing behavior, and it can be fixed under a normal optical microscope objective. However, the optical microscope doesn’t have any quantitative measurement capacity. In subsequent research, Tyrrell developed an integrated head combining an optical interference objective and an AFM [7].

Besides the mentioned shortcomings, the measuring range of the AFM is generally limited to 100 μm × 100 μm × 10 μm. In this regard, one method to expand the AFM’s measuring range is designing new scanners with larger scanning range, but because of the limitations of the scanner’s mechanical structure and the piezo actuators’ properties, the range of the scanner can hardly exceed hundreds of micrometers [8]. Another method is to combine an AFM with a large-range positioning system [9,10]. In this method, laser interferometers are intended to guarantee both measurement accuracy and traceability in a large-range.

In this paper, a hybrid AFM measurement system combined with white light scanning interferometry is developed. The compact self-sensing AFM head doesn’t need optical sensors to detect the deflection of the cantilever, and can be directly assembled under an optical interference system. A hybrid measuring method combining white light scanning interferometry is proposed to enhance AFM’s measuring efficiency. Based on a high precision large range positioning platform, the lateral scanning range of the system can reach tens of millimeters and the measurement data can be traced to the meter definition through embedded laser interferometers.

## 2. System Design

**Figure 1.**
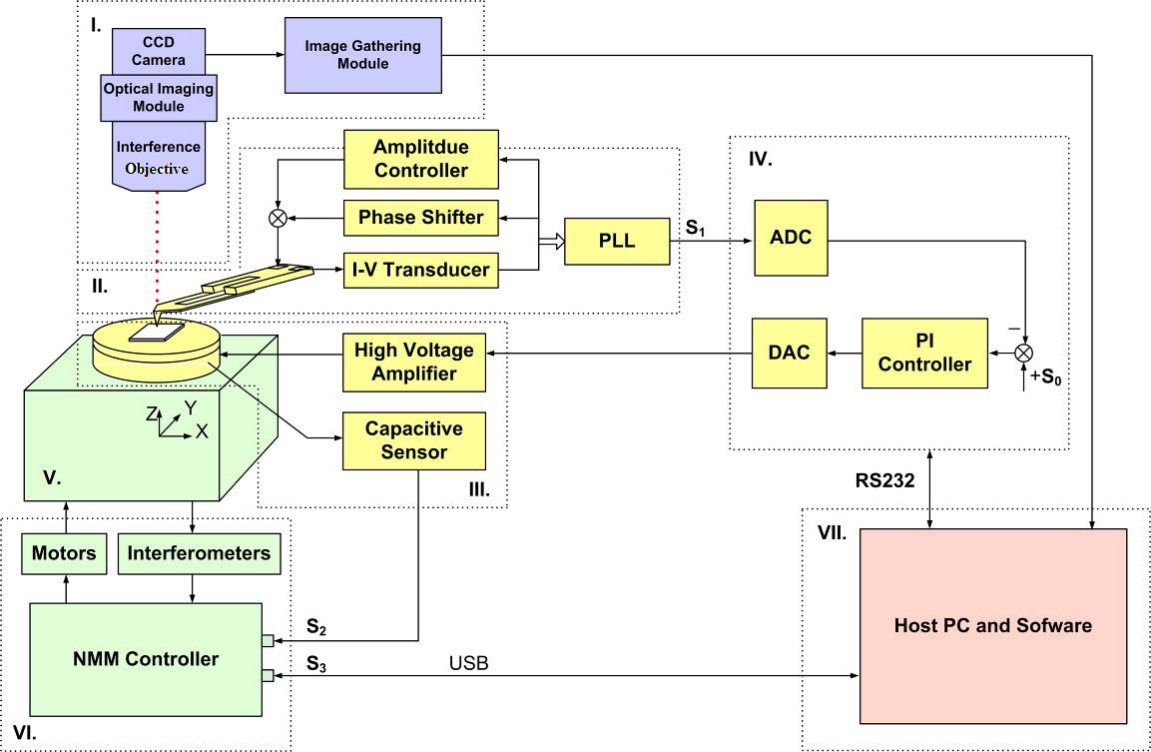
The schematic diagram of the hybrid AFM measurement system.

The hybrid AFM measurement system consists of seven main blocks (see Figure 1):

- *Optical interference measurement module.* This module can be used as a normal optical microscope to real-time monitor the probe, sample and measurement conditions. More significantly, by installing an interference objective, it can be set as a white light scanning interferometer to be used in combination with the AFM.
- *AFM measuring head and frequency modulation (FM) module*. The AFM head uses a self-sensing tuning fork probe and can translate the resonant frequency change of the probe during the measurement into a voltage output with the FM module.
- *Z-axis positioning stage driven by a piezoelectric ceramic actuator (PZT)*. It moves in the z-axis with a stroke of 2 μm, and has a built-in capacitive sensor to detect the displacement.
- *High speed digital signal processing (DSP) servo feedback control system*. This system can control the movement of PZT stage and maintain the distance between the tip and the sample at a constant value.
- *Large-range nanometer positioning platform*. It can realize the x-, y- and z-axis movement within the range of 25 mm × 25 mm × 5 mm.
- *NMM controller and three axis laser interferometers*. NMM controller controls the motion of the NMM in close loop. The interferometers guarantee the traceability of system’s positioning and measurement.
- *Host PC and measurement software*. The PC has three main functions. Firstly, it can record imaging data of the optical measurement system. Secondly, it can communicate with the DSP module through a RS232 interface, and adjust various parameters of the feedback controlling algorithm. Thirdly, it can communicate with the NMM through an USB interface to set the parameters of the NMM, and collect the measurement results. S_0_ is the setpoint regarding the tip-sample interaction force; S_1_ is the senor signal from the FM module; S_2_ is the extension value of the PZT stage; S_3_ are measurement commands and data.

The hybrid measurement system is based on the large-range nanometer positioning platform. The nano-measuring machine (NMM) produced by SIOS Messtechnik GmbH [11] is used as the large-range nanometer positioning platform. The main structure of the displacement platform is made up of three orthogonal Zerodur® plane mirrors with very low coefficient of thermal expansion (α = 0 ± 0.15 μm k^−1^). The accuracy of the mirrors’ reflective surfaces is λ/20. The orthogonality deviations are better than 2”. There are three built-in laser interferometers in the NMM capable to reach a 0.1 nm measuring resolution and trace the measurement data to the meter definition. There are two 0.1” resolution angle sensors in the x and y axes to compensate the angle deflection of the platform in collaboration with the NMM controller.

**Figure 2.**
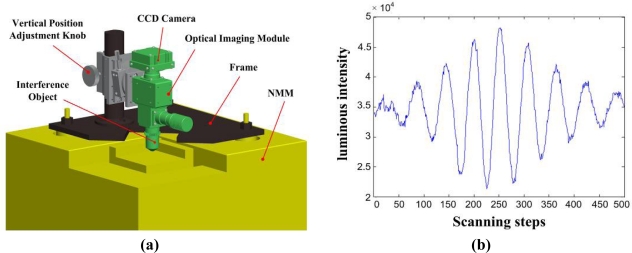
**(a)** The optical interference measuring module and **(b)** white light interference signal.

The optical interference microscopic module is fixed on the frame of the NMM, as depicted in Figure 2(a). The optical interference module is made of five parts: illuminator, interference objective, optical imaging module, CCD camera and image acquisition module. The halogen illuminator manufactured by Nikon was selected to provide a good lighting environment. The fiber transducer was selected to reduce the thermal radiation disturbance. In order to get a wide field of view, Nikon 5× and 10× interferometric objectives with a working distance of 9.3 mm and 7.4 mm, respectively, were selected, which provide enough space to install the AFM head. The image acquisition module uses a vertical illumination microscopic tube (CM30A) made by Nikon. The CCD camera (A102k) uses a monochromatic digital camera produced by Basler. An image acquisition card with a cameralink interface (NI 1429) from NI company is used to transfer images from the CCD camera to the PC. To realize the interference measurement, the NMM is selected as the vertical movement mechanism to realize a 5 mm measuring range travel in the vertical direction. The optical measuring method uses white light scanning interferometry which is based on two beams interference theory. Due to the fact white light has a wideband spectrum, all wavelength components will present interference separately in the spectrum. The non-coherent overlap of interference images from various wavelength components forms the white light interference signal as shown in Figure 2(b). When the light path difference between two interference beams is zero, interference signal will reach the maximum or minimum value. The position of interference peak represents the height information of structure surfaces.

**Figure 3.**
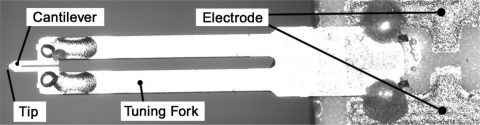
The photograpgy of tuning fork probe.

In order to use the optical measuring methods, a compact AFM head is required. Moreover, the contact force between the tip and the sample should be minimized to reduce the damage on sample surface made by the probe tip. Hence, in order to meet the aforementioned requirements, a novel tuning fork probe is adopted [12,13]. This kind of probe is invented by Neuchâtel University and has been commercialized by Nanosensors Corporation. As shown in Figure 3, a U-shaped silicon nitride cantilever with a probing tip at its free end is combined in a symmetrical arrangement with a quartz tuning fork such that each of the two legs of the cantilever is fixed to one of the two prongs of the tuning fork. The parameters of the AFM probe are as follows: the resonant frequency is 45–55 kHz; the spring constant is about 5 N/m; the length is 310 μm; the width is 30 μm; the thickness is 3.7 μm; the diameter of the tip is below 15 nm. The tip points to the vertical direction, perpendicular to lateral plane defined by the tuning fork and cantilever. Under the action of the tuning fork, the probe has a self-sensing behavior and can convert the deflection of cantilever to a voltage change. Because of the probe’s unique structure, the probe can be benefited from both the tuning fork’s high quality factor [14,15] and a extremely stable oscillation and the silicon cantilever's reasonable spring constant which will produce a smaller tip-sample contact force.

Since the current signal in the tuning fork probe is so weak (nA range), an I-V transducer is used to amplify the weak signal with a 10^7^ magnification, as shown in Figure 1. Due to the probe has a high quality factor (>500), which will limit the dynamic response of the AFM head in amplitude modulation, the FM operating mode is adopted for the head. In the FM mode, the sample surfaces’ topography is characterized by the change of the probe’s resonant frequency. As shown in Figure 1, the FM module is composed of amplitude controller, phase shifter and phase-locked loop (PLL). The amplitude controller and the phase shifter can adjust the amplitude and phase of the signal in the circuit loop to form the probe self-excitation oscillation. The PLL can convert the resonant frequency into a voltage value. In the measurement, the frequency change in the circuit loop is input to the PLL which detects the frequency change of the reflecting sample surfaces [16].

**Figure 4.**
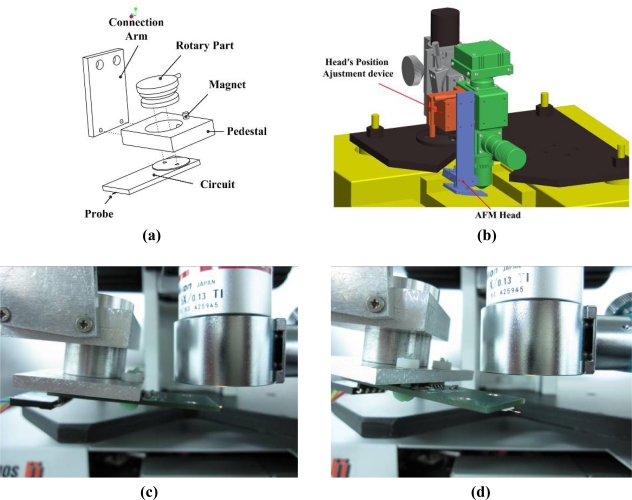
**(a)** The AFM head’s mechanical structure, **(b)** assembly drawing, **(c)** the working position, and **(d)** the position removed out from the field of view.

Based on the self-sensing tuning fork probe, the AFM head doesn’t need external optical sensors and this saves room on the head. The compact rotary head’s mechanism is designed for real-time observation of the measurement situation, and for the combination of the AFM head and the optical measuring method. As depicted in Figure 4(a), the head’s mechanism is made up of four parts: circuit, rotary part, pedestal and connection arm. Through the holder, the probe is fixed on the circuit assembled under the rotary part. The rotary part is settled in the center holes of the pedestal to provide the probe a rotational motion. As shown in Figure 4(b,c), the connection arm can fix the head under the optical interference module. The function of the rotational motion is to move the probe out of the field of the optical module’s view to not block optical measurement. The magnet on the pedestal enhances the fixation and positioning precision when the probe restores its initial position, as shown in Figure 4(d). To reduce measurement errors, the system is designed following the Abbe principle. The measuring axes of the interferometers intersect at the contact point of the AFM head.

## 3. Calibration and Measurement Experiments

### 3.1. Calibration of the PZT Stage

**Figure 5.**
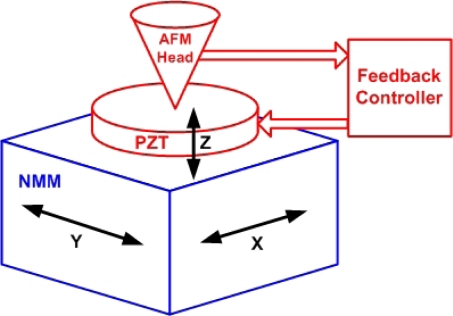
The measuring mode of the AFM system.

The measuring mode of the system is shown in Figure 5. The sample is placed on the PZT stage (from PI, s303). The NMM platform moves the sample and the PZT stage moving in the x-y plane, therefore the scanning range can reach millimeter scale. In the scanning process, the AFM head detects the sample surface’s height information point by point. The PZT stage is controlled by a feedback controller to move it in the vertical direction, keeping the distance between the tip and the sample at a constant value. The displacement in the lateral direction is directly recorded by the laser interferometers embedded in the NMM, and can be traced to the meter definition. The height information in the vertical direction is detected by the capacitive sensor in the PZT stage, and it needs to be calibrated in advance to achieve traceable topographic measurements.

**Figure 6.**
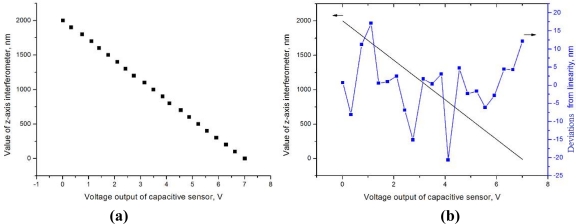
**(a)** The data collected during the calibration of the PZT stage’s capacitive sensor and **(b)** the calibration results.

For this purpose, the method proposed to calibrate the capacitive sensor against the z-axis interferometer of the NMM is the following: after approaching the tip towards the sample, the fast feedback loop is activated so that bending of the cantilever is kept constant by the PZT stage; while keeping the NMM’s lateral positions unchanged, the sample is then moved upward in the vertical direction by the NMM; therefore, the PZT stage moves down and the sum value of the z-axis interferometer and the capacitive sensor should be constant; by recording simultaneously the value of the z-axis interferometer and capacitive sensor, the capacitive sensor can be calibrated through the calibration equation as follow as depicted in Figure 6(a).

In the Equation (1), *A* is the extension of PZT detected from the z-axis interferometer; *u_c_* is the output of capacitive sensor; *kaz* [0⋯3] are the calibration parameters. Because the *A* and *u_c_* have linear relationship, *kaz* [2, 3] are set as zero to linearly fit the data with a least squares method. Figure 6(b) shows the calibration results. The left Y axis is the fitted straight-line with the slope *kaz* [1] = −208 nm/V, which represents the capacitive sensor’s sensitivity, which is closed to the design value (−200 nm/V). The right Y axis observes deviations from linearity. The linearity deviation, which is less than 1%, is contributed by the nonlinear response of the interferometer and the capacitive sensor, in addition to the overall noise of the system:

(1) $$A=\mathrm{kaz}[0]+\mathrm{kaz}[1]\cdot u_{c}+\mathrm{kaz}[2]\cdot u_{c}^{2}+\mathrm{kaz}[3]\cdot u_{c}^{3}$$

### 3.2. AFM Measurement Experiment

**Figure 7.**
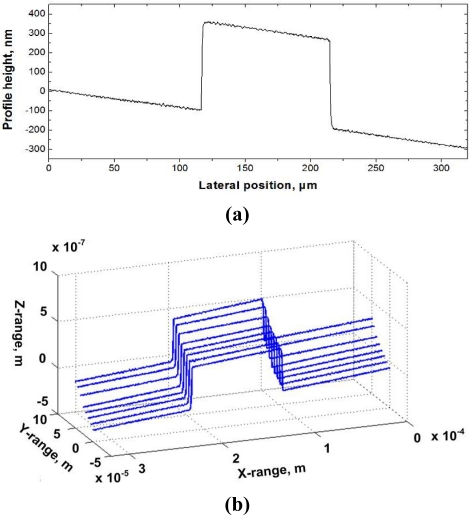
The cross section profile **(a)** and multiple scanning measured results **(b)** of 450 nm step standard.

To test the measuring performance of the AFM system, a step standard sample is measured. The standard step height is produced by VLSI Corporation. The substrate material is quartz with dimensions of 25 mm × 25 mm × 3 mm and the step height material is made of silicon dioxide with a height of 454.8 ± 2.7 nm calibrated by NIST. The measurement results in Figure 7(a) show a scanning length of 160 μm and scanning speed of 1 μm/s. For evaluation, the sample is measured 8 times in the range of 320 μm × 50 μm. Figure 7(b) shows the measurement results.

The evaluation of the step height standard is according to ISO5436-1: 2000. The measurement data needs to be leveled before being evaluated. The evaluation results are shown in Table 1. As shown in Table 1, the measured average value is 454.3 nm and the standard deviation is 0.5 nm. The results demonstrate good measurement precision and repeatability of the system.

### 3.3. Hybrid Measurement Experiment

The lateral measurement range of the AFM system can reach millimeter scale; however, the measuring efficiency is limited as a result of the point-to-point scanning approach. Consequently, the optical microscopic measuring method is used to complement the AFM to realize hybrid measurement.

Regarding the benefits of the optical measuring method, on the one hand, due to the use of the field measuring mode, the measuring efficiency of white light scanning interferometry is much higher than that of the AFM; on the other hand, white light scanning interferometry belongs to the non-contact measurement methods, providing pre-inspection of the sample to avoid dangerous areas for the AFM system.

Based on the features of the large-range positioning platform, a hybrid measuring method combing the AFM and the white light scanning interferometry is proposed in order to enhance the measuring efficiency of the AFM by means of the localization of white light scanning interferometry. The basic procedure is to use white light scanning interferometry to conduct quick measurements on sample surfaces with a relatively low lateral resolution. Then, according to the measuring results of the white light scanning interferometry the regions of interest (ROI) on the sample are chosen. The dangerous region which is beyond the AFM system’s measuring ability can also be found and excluded from the ROI. Finally, the selected ROI is moved to the position under the AFM head by the NMM and then measured more accurately by the AFM. The method can not only improve the measuring efficiency of the AFM but also protect AFM head.

**Figure 8.**
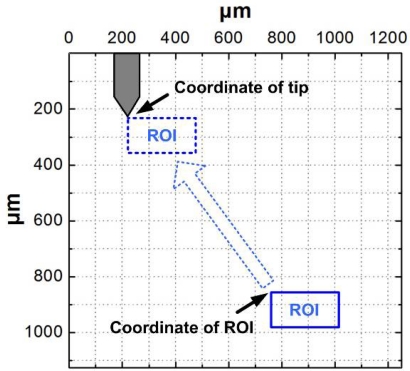
The coordinate unified schematic drawing of the AFM and the white light scanning interferometer.

A critical problem to be solved is how to unify the measuring coordinate system of the AFM and the white light scanning interferometer. The white light scanning interferometer can be used as a normal optical microscope which has the same lateral coordinate system determined by the magnification of objective. Hence, the solution is to unify the AFM plane coordinates into the white light scanning interferometer’s imaging coordinate system. As depicted in Figure 8, when the white light scanning interferometer get the images of the probe and sample surface, the lateral coordinates of which become the pixel position in the CCD camera. From this, the coordinate deviation between the probe’s tip and the ROI can be calculated. Then, the ROI is moved to the position of tip by the NMM and then can be measured by the AFM. The NMM has a 0.1 nm lateral resolution which is much smaller than the optical system’s, meeting the positioning requirements.

**Figure 9.**
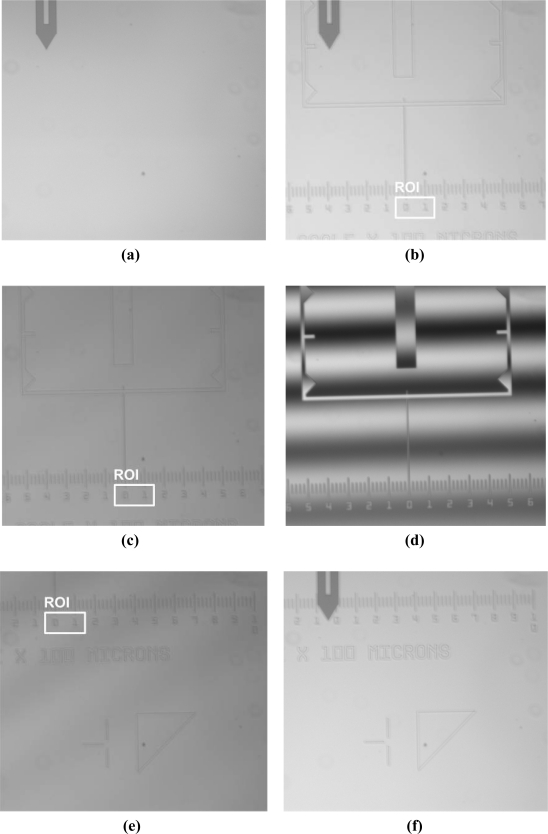
The experimental procedures of the hybrid measuring methods.

**Figure 10.**
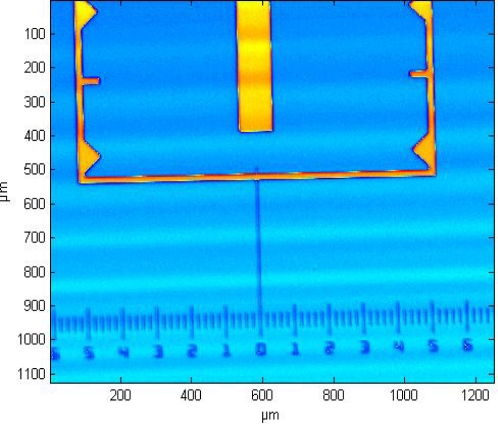
The measurement results by the white light scanning interferometry.

**Figure 11.**
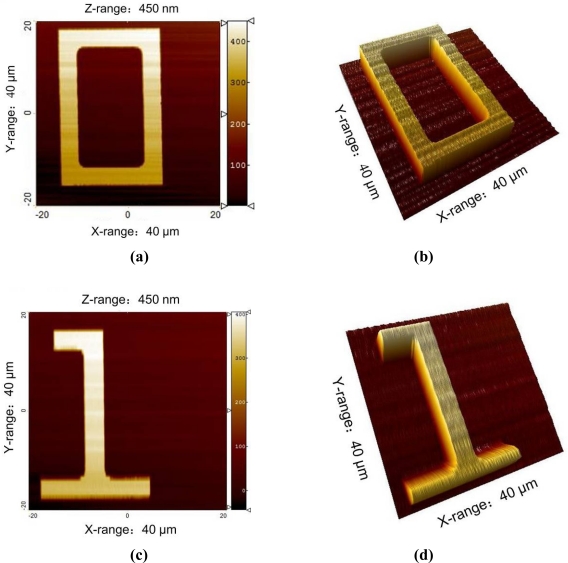
The measurement results of the ROI’s two regions by AFM: **(a)** and **(c)** are the two-dimensional images; **(b)** and **(d)** are the three-dimensional images.

In the experiment, a 5× objective is selected to collect more topography information of the sample surface. The imaging area of CCD is 1,000 pixel × 900 pixel corresponding to 1,250 μm × 1,125 μm in the measuring coordinate system which means that the pixel length is 1.25 μm. The white light interference peak is determined by using the center of gravity method. By adjusting the NMM, the length of vertical scanning step is set as 50 nm, and the number of steps is set as 140 corresponding to a 7 μm scanning range. The measuring process of the white light scanning interferometry takes about 2 minutes. The specific experimental procedures are as follows:

1. As shown in Figure 9(a), while keeping the sample far from the tip, the distance between the probe and the objective is adjusted to clearly image the probe in the field of view. A plane sample, like a silicon wafer, is used to make a monochrome background. The top-left corner of the field of view is set as the origin of coordinate. The lateral pixel coordinate of the end of cantilever is (176, 194) corresponding to (220 μm, 242.5 μm).
2. The 450 nm step height standard is chosen to perform measurement experiments. The NMM platform is moved precisely to make the sample imaged in the field of view. Then, the ROI is selected, as shown in Figure 9(b). In order not to disturb the white light scanning interferometer, the cantilever is moved out of the field of view through the head’s rotary part, as depicted in Figure 9(c).
3. The sample is measured by the white light scanning interferometer. In the measurement, moving interference fringes can be observed, as shown in Figure 9(d). The measurement result is shown in Figure 10, in which the coordinate of the ROI’s top-left corner is (560 μm, 987.5 μm). From the results, the height change of the ROI is smaller than 500 nm which means that it is suitable for the AFM measurement.
4. The sample is moved in lateral directions by the NMM until the position of the top-left of the ROI coincides with the one of the cantilever’s end, as shown in Figure 9(e).
5. As depicted in Figure 9(f), the cantilever is moved back to its initial position. Two 40 μm × 40 μm regions of ROI are additionally measured by the AFM. The measurement results are shown in Figure 11.

## 4. Conclusions

A hybrid AFM measurement system combined with white light scanning interferometry based on a precise large range positioning platform is developed. The AFM head has a self-sensing behavior and can be conveniently fixed under the objective of the optical interference system. The FM operation mode is adopted to overcome the dynamic response limitation by using a probe’s high quality factor. The AFM head is set up on a large-range positioning platform capable of performing millimeter scale lateral scanning. The measurement of a step standard demonstrates a good measurement accuracy and repeatability of the system. The measurement experiments illustrated that the efficiency of the AFM can be improved through this method.

## Figures and Tables

**Table 1.** The evaluation results of 450 nm step height standard.

**No.**	**Height(nm)**	**Average value(nm)**	**Standard deviation (nm)**
1	454.3		
2	453.7		
3	453.8	454.3	0.50
4	454.7		
5	454.8		
